# Spinal Implant–Associated Infection in Type 2 and Type 1 Diabetes: Phenotype‐Specific Inflammatory Features and Therapeutic Response to Semaglutide

**DOI:** 10.1002/jsp2.70196

**Published:** 2026-05-28

**Authors:** Thomas E. Olson, Trevor S. Lloyd, Christopher D. Hamad, Rene F. Chun, Joshua Wiener, Autreen Golzar, Joshua Mehany, Soroush Shahamatdar, Andrew Kittredge, Lauren Pearce, Kevin P. Francis, Farres Obeidin, Langston T. Holly, Michael R. Yeaman, John S. Adams, Nicholas M. Bernthal, William L. Sheppard

**Affiliations:** ^1^ Department of Orthopaedic Surgery David Geffen School of Medicine at UCLA Santa Monica California USA; ^2^ Case Western Reserve University School of Medicine Cleveland Ohio USA; ^3^ University of California, Los Angeles Los Angeles California USA; ^4^ Department of Pathology and Laboratory Medicine David Geffen School of Medicine at UCLA Los Angeles California USA; ^5^ Department of Neurosurgery David Geffen School of Medicine at UCLA Los Angeles California USA

**Keywords:** cytokine profile, immune dysregulation, metabolic intervention, murine model, spinal implant‐associated infection, Type 1 diabetes mellitus, Type 2 diabetes mellitus, wound healing

## Abstract

**Background:**

Diabetes mellitus (DM) is a major risk factor for postoperative infection and wound complications in spine surgery, yet distinctions between Type 2 (T2DM) and Type 1 (T1DM) pathophysiology are rarely addressed. This study compares infectious burden, wound healing, and immune response among a murine model of spinal implant‐associated infection of T2DM, T1DM, and nondiabetic control mice before and after metabolic intervention with the GLP‐1 receptor agonist (GLP‐1RA), semaglutide.

**Methods:**

Male C57BL/6J mice were rendered diabetic by streptozotocin induction (T1DM) or a high‐fat, high‐sucrose diet (T2DM). Spinal implants were placed and inoculated with bioluminescent *Staphylococcus aureus* . Infection progression was monitored longitudinally by in vivo bioluminescence imaging. Systemic inflammation was characterized through multiplex cytokine profiling, and paraspinal tissues were analyzed by histology and multiplex immunofluorescence.

**Results:**

Both diabetic models demonstrated greater infection burden, delayed wound healing, and distinct systemic inflammatory profiles compared with controls. T2DM was characterized by chronically elevated baseline inflammation and a blunted acute response to infection, whereas T1DM exhibited low baseline activity but exaggerated and dysregulated cytokine induction. Semaglutide attenuated infection severity, improved wound integrity, and partially normalized inflammatory patterns. Histology and immunofluorescence corroborated these findings, showing reduced immune cell infiltration and improved tissue organization in semaglutide‐treated cohorts.

**Conclusions:**

T2DM and T1DM are associated with differing inflammatory and immune features in murine spinal implant–associated infection. Metabolic modulation with semaglutide restores immune balance, reduces infection severity, and promotes wound repair. These findings support exploration of GLP‐1RA‐based therapies to improve surgical outcomes in diabetic patients.

## 1 Introduction

Diabetes mellitus is a growing global epidemic and a well‐established risk factor for complications following orthopedic and spine surgery [1, 2, 3]. Patients with diabetes experience higher rates of surgical site infection, delayed wound healing, and hardware failure compared to non‐diabetic individuals [2, 3]. These risks are particularly pronounced in the context of spine surgery and orthopedic implant procedures, where infection can result in devastating morbidity, revision surgery, and prolonged disability.

Despite the clear association between diabetes, obesity, and adverse surgical outcomes, most existing clinical studies have grouped all forms of diabetes mellitus together, often relying on preoperative hemoglobin A1c (HbA1c) levels as the primary metric of disease severity and glycemic control [4, 5, 6, 7, 8]. While elevated HbA1c is a useful indicator of chronic hyperglycemia and is associated with increased perioperative risk, this approach overlooks critical differences in the underlying pathophysiology of Type 1 diabetes mellitus (T1DM) and Type 2 diabetes mellitus (T2DM) [9, 10, 11, 12].

T1DM is characterized by autoimmune‐mediated destruction of pancreatic beta cells, resulting in absolute insulin deficiency and a catabolic state [13]. In contrast, T2DM is driven by a combination of peripheral insulin resistance and relative insulin deficiency, typically associated with obesity, chronic low‐grade inflammation, and metabolic syndrome [14, 15]. These fundamental distinctions influence not only glycemic patterns, but also immune competence, inflammatory tone, and host response to infection [9, 16]. However, there is a relative paucity of data examining how these unique pathologies impact infection and wound healing in the setting of orthopedic and spine surgery.

Recent advances in pharmacologic management of diabetes, including the use of glucagon‐like peptide‐1 receptor agonists (GLP‐1 RAs) such as semaglutide, have demonstrated potent effects on glycemic control, weight reduction, and systemic inflammation [17, 18]. These medications are now ubiquitously used within T2DM treatment and increasingly utilized in the setting of obesity and metabolic syndrome. While GLP‐1 RAs are primarily approved for T2DM, emerging data suggest that GLP‐1 signaling also influences immune and metabolic pathways relevant in both T1DM and T2DM, and are occasionally used to aid in diabetic control for patients with T1DM [19, 20]. In T2DM, semaglutide improves insulin sensitivity, reduces adipose‐driven inflammation, and promotes metabolic homeostasis, whereas in T1DM, preclinical studies suggest potential immunomodulatory effects that may mitigate chronic inflammation and support tissue repair [21]. Collectively, these findings indicate that GLP‐1‐based therapy may exert beneficial effects beyond glucose regulation, potentially improving host defense and wound healing after surgical insult. Despite this, there is little to no preclinical data evaluating the effects of GLP‐1 RAs on infection and wound healing by distinct diabetic subtypes in the context of orthopedic implants.

The role of diabetes and GLP‐1‐based therapy has been identified as a high‐priority research topic in musculoskeletal and implant‐associated infections [22, 23]. This study aims to address this growing area of interest by directly comparing the effects of T2DM and T1DM on surgical site infection, wound healing, and immune response in a controlled murine model of spinal implant‐associated infection. To test this, we developed a spinal implant–associated infection model in murine T2DM and T1DM, validated that each model phenocopies the human disease, and examined how semaglutide alters glycemic control, infection burden, wound healing, and systemic and local immune responses. We hypothesized that T2DM and T1DM create distinct immuno‐metabolic environments that impair host defense against spinal implant infection, and that pharmacologic GLP‐1 receptor activation with semaglutide could partially normalize these states. By distinguishing between these two forms of diabetes and interrogating their interactions with semaglutide, we seek to provide insight that may inform more personalized perioperative risk assessment and therapeutic strategies for diabetic patients undergoing spine and orthopedic procedures.

## 2 Materials and Methods

### 2.1 Animal Models and Induction of Diabetes

All animal experiments were performed in accordance with the Institutional Animal Care and Use Committee (IACUC) guidelines (ARC‐2012‐104). Male C57BL/6J mice (Jackson Laboratories) were acclimated for at least 1 week prior to study enrollment. Mice were housed under standard conditions with ad libitum access to food and water and maintained on a 12‐h light/dark cycle.

T2DM with concomitant obesity was induced by feeding mice a high‐fat, high‐sucrose diet (Research Diets, #D03062301, 60% kcal from fat with increased sucrose) for a minimum of 8 weeks prior to surgery. Body weight and fasting glucose were monitored weekly. Mice were considered diabetic if fasting glucose exceeded 200 mg/dL and demonstrated impaired glucose clearance on tolerance testing. Age‐matched mice fed with standard chow served as non‐diabetic controls.

As a glycemic control, T1DM was induced by intraperitoneal injection of streptozotocin (STZ; Fisher Scientific, #AAJ61601ME) at a dose of 40 mg/kg body weight dissolved in citrate buffer (pH 4.5), administered daily for five consecutive days. Blood glucose levels were monitored from the tail vein weekly following injection. Mice were considered diabetic if fasting glucose was consistently > 200 mg/dL.

Glucose tolerance tests were performed after a 6‐h fast. Mice received an oral gavage bolus of D‐glucose (2 g/kg body weight), and blood glucose was measured from the tail vein at baseline (0 min), and at 15, 30, 60, and 120 min postinjection using a calibrated handheld glucometer (Germaine Laboratories AimStrip Plus Blood Glucose Testing System, Fisher Scientific). Fasting, peak, and 120‐min glucose levels were calculated.

A total of 134 male C57BL/6J mice were allocated across 10 experimental groups. Infected groups included nondiabetic controls (*n* = 10), T2DM (*n* = 27), T2DM + semaglutide (*n* = 15), T1DM (*n* = 27), and T1DM + semaglutide (*n* = 15). Corresponding sterile controls included nondiabetic (*n* = 10), T2DM (*n* = 10), T2DM + semaglutide (*n* = 5), T1DM (*n* = 10), and T1DM + semaglutide (*n* = 5).

### 2.2 Surgical Model of Spinal Implant‐Associated Infection

All surgical procedures were performed under aseptic conditions. C57Bl/6J mice at 12 to 14 weeks of age were anesthetized with isoflurane (1%–3%) in oxygen. The dorsum was shaved and sequentially prepped with betadine and alcohol before being placed prone on a sterile field. In accordance with the prior published model [24, 25], a 2.0 cm midline dorsal skin incision was made to expose and dissect the lumbar paraspinal musculature from the underlying vertebrae. A 0.25 mm diameter 10 mm length custom L‐shaped stainless‐steel wire (Modern Grinding, Port Washington, Wisconsin, USA) was placed through the exposed spinous process between the L3–L5 levels (Figure S1).

For infection groups, the implant was inoculated intraoperatively with 1E3 CFU of bioluminescent *Staphylococcus aureus* (Xen36, Revvity, 119243, Hopkinton, Massachusetts, USA) suspended in 2.4 μL phosphate‐buffered saline (PBS). Sterile control groups received implants without bacterial inoculation. The incision was closed with layered 5–0 absorbable sutures, and animals were monitored until full recovery from anesthesia.

### 2.3 Bioluminescence Imaging, Infection Monitoring, and Bacterial Quantification (CFU Counting)

To assess infectious burden, mice were imaged at baseline and for 6 weeks postoperatively using an IVIS Spectrum optical imaging system and Living Image software (Revvity). Mice were anesthetized with isoflurane and placed prone in the imaging chamber. Standardized regions of interest (ROIs) encompassing the surgical site were drawn, and total photon flux (photons/s) was quantified (Figure S2). Mice were imaged for anterior–posterior and lateral projection X‐rays using the IVIS Lumina X5 (Revvity) on postoperative days (POD) 0, 7, 21, 35, and 42 (Figure S1).

Wounds were monitored daily for evidence of dehiscence, erythema, or discharge. Wound dehiscence was defined as visible separation of the skin incision with exposure of the underlying fascia, deep tissues, and/or implant. The day of first wound dehiscence was recorded for Kaplan–Meier survival analysis. Animals that experienced wound dehiscence were euthanized at the time of the event; data collected prior to dehiscence were included in all preceding time‐point analyzes. No spontaneous deaths occurred during the study period.

At postoperative day 42, mice were euthanized by isoflurane inhalation followed by cervical dislocation. Implants and surrounding peri‐implant tissues were aseptically harvested. Tissue was weighed and homogenized in sterile PBS using a tissue homogenizer (Pro200 Series homogenizer, Pro Scientific). Bacteria adherent to implants were detached by vortex and sonication in 250 μL of 0.4% tween in tryptic soy broth for 10 min. Serial dilutions of implant and tissue samples were performed in a 96‐well plate prior to plating on tryptic soy agar plates, followed by overnight incubation at 37°C. CFUs were counted after 18 h and normalized to tissue weight (CFU/g) or reported per implant. Positive and negative control plates were included in each batch.

### 2.4 Semaglutide Rescue Intervention

A subset of each T2DM and T1DM cohort received semaglutide (Adipogen Corporation, #AG‐CP3‐0040) administered by subcutaneous injection at a dose of 30 nmol/kg daily, starting 14 days before surgery and continuing through the postoperative period and until conclusion of the experiment. Glucose tolerance, body weight, infection burden, and wound outcomes were monitored as above.

### 2.5 Serum Collection, Cytokine Analysis, and Tissue Sampling

At the study endpoint, immediately following euthanasia, fresh whole blood was collected by terminal cardiac puncture using a presiliconized, 25‐gage needle and syringe and stored in EDTA tubes. Samples were preserved on ice and sent for outside laboratory analysis (IDEXX BioAnalytics, West Sacramento, California, USA). Panels included CBC (IDEXX BioAnalytics, #61330), C‐Reactive Protein (IDEXX BioAnalytics, #62458), and Cytokine Mouse 25‐Plex (IDEXX BioAnalytics, #62579). Cytokine, blood, and differential concentrations were analyzed as both absolute values and heatmap representations.

Tissue samples were subsequently harvested, and formalin‐fixed, decalcified mouse spine specimens were processed and paraffin‐embedded. Sections were prepared for both hematoxylin and eosin (H&E) staining and multiplex immunofluorescence (mIF) analysis. H&E staining was performed using standard histopathologic protocols to evaluate overall tissue morphology, fibrosis, and inflammatory cell infiltration. For mIF, deparaffinized sections underwent antigen retrieval, blocking, and sequential incubation with primary antibodies followed by fluorophore‐conjugated detection. The staining panel included DAPI (nuclear counterstain), Ly6G (neutrophils), CD20 (B cells), CD45 (pan‐leukocyte marker), F4/80 (macrophages), and CD3 (T cells). Panel development followed core‐laboratory optimization procedures to confirm signal specificity and channel separation, with appropriate positive and negative control slides processed in parallel. After staining, whole slides were scanned, and multichannel images were delivered for downstream visualization and quantitative review. Image assessment was performed using full‐resolution multiplex files, with individual channels and composite overlays examined to evaluate staining distribution and cellular localization.

### 2.6 Data Management and Statistical Analysis

Sample size calculations were performed a priori to detect expected differences in infectious burden and wound healing based on preliminary data. All data were entered into a secure database and verified for accuracy. Statistical analyzes were performed using Python (v3.1, Python Software Foundation) or R (v4.5, R Core Foundation). Continuous variables were assessed for normality using the Shapiro–Wilk test and compared using one‐way ANOVA or Kruskal–Wallis tests, with appropriate post hoc corrections (Tukey's or Dunn's test). Categorical variables were compared using the chi‐squared test or Fisher's exact test. Kaplan–Meier survival curves were compared using the log‐rank (Mantel‐Cox) test. A *p*‐value < 0.05 was considered statistically significant.

## 3 Results

### 3.1 Phenocopying Human T2DM and T1DM in Mouse Model

#### 3.1.1 Body Weight Trajectories

**FIGURE 1:**
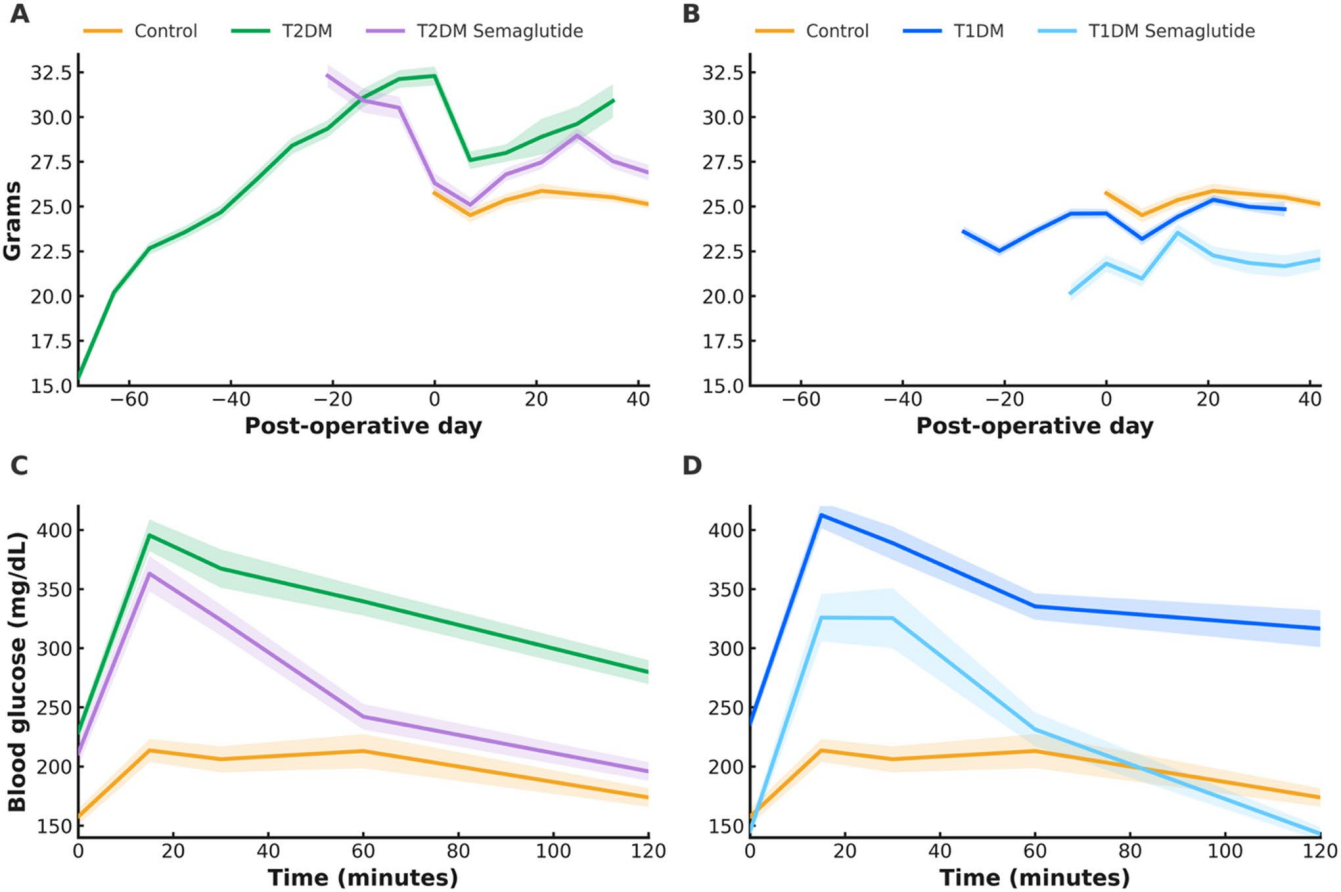
Perioperative body weight trajectories and glucose tolerance testing (GTT) in murine diabetes models. (A) Body weight curves for nondiabetic (control) and Type 2 diabetic (T2DM) mice, with and without semaglutide, during the treatment and perioperative period. (B) Body weight curves for control and Type 1 diabetic (T1DM) mice (with and without semaglutide). On the day of surgery, mean body weight differed significantly across groups (control: 25.7 ± 0.3 g; T2DM: 32.3 ± 0.5 g; T1DM: 24.6 ± 0.3 g; *p* < 0.001). Semaglutide reduced body weight by 14.9% in T2DM mice to a level indistinguishable from controls (*p* < 0.001). (C) Fasting and postglucose challenge blood glucose levels over 120 min in control, T2DM, and T2DM + semaglutide mice. (D) Fasting and post‐glucose challenge blood glucose levels over 120 min in control, T1DM, and T1DM + semaglutide mice. GTT was performed 1 day prior to surgery after a 6‐h fast with oral glucose gavage (2 g/kg). Fasting glucose was significantly elevated in T1DM (236.4 ± 7.3 mg/dL) and T2DM (228.9 ± 7.1 mg/dL) compared to control (157.6 ± 6.8 mg/dL; *p* < 0.001). Peak postchallenge glucose was higher in T1DM (395.4 ± 13.4 mg/dL) and T2DM (412.5 ± 11.0 mg/dL) versus control (*p* < 0.001). Semaglutide reduced AUC by 32.2% in T1DM (*p* < 0.001) and 21.5% in T2DM (*p* < 0.001) compared to untreated diabetic counterparts. Data represented as mean ± SD (bold line) within the shaded range. N: Non‐diabetic control = 20, T2DM = 37, T2DM + semaglutide = 20, T1DM = 37, T1DM + semaglutide = 20. Statistical comparisons by one‐way ANOVA with Tukey's post hoc correction.

On the day of surgery, T2DM mice exhibited the highest mean body weight (32.3 ± 0.5 g), consistent with obesity and metabolic syndrome, while control mice averaged 25.7 ± 0.3 g, and T1DM mice had the lowest mean body weight at 24.6 ± 0.3 g (*p* < 0.001; Figure 1A,B). Following surgery, all groups experienced a notable decline in weight during the first 7 postoperative days, reflecting the physiological stress of surgery and infection. T2DM mice showed the greatest absolute decrease in weight, while control and T1DM mice experienced more modest losses (Figure 1A,B). After this initial postoperative period, all groups demonstrated recovery of body weight, with weights stabilizing or trending upward through the remainder of the observation period. Semaglutide treatment beginning 14 days preoperatively reduced body weight in both T1DM and T2DM cohorts compared to untreated diabetic counterparts. In the T2DM group, weight was reduced by 14.9% to a level that was indistinguishable from nondiabetic controls and attenuated perioperative weight loss following surgery (*p* < 0.001; Figure 1B).

#### 3.1.2 Glucose Tolerance Test (GTT)

Murine models of Type 1 diabetes mellitus (T1DM) and Type 2 diabetes mellitus (T2DM) were successfully established using streptozotocin induction and a high‐fat, high‐sucrose diet, respectively. Validation of these models was confirmed by glucose tolerance testing. Fasting glucose was elevated in both T1DM (236.4 ± 7.3 mg/dL) and T2DM (228.9 ± 7.1 mg/dL) compared to control (157.6 ± 6.8 mg/dL; *p* < 0.001; Figure 1C,D). Diabetic mice exhibited significantly higher peak blood glucose levels (T1DM: 395.4 ± 13.4 mg/dL; T2DM: 412.5 ± 11.0 mg/dL) following glucose challenge compared to nondiabetic controls (*p* < 0.001; Figure 1C,D). In T1DM mice, blood glucose remained elevated throughout the 120‐min time course, with values elevated 37% ± 7% over fasting glucose levels at 120 min. T2DM mice also demonstrated impaired glucose clearance, though a gradual reduction was observed with a return to 26% ± 5% over fasting glucose, consistent with insulin resistance rather than absolute deficiency. Control mice displayed a modest rise in glucose with 11% ± 4% elevated fasting glucose by 120 min (Figure 1).

Administration of semaglutide in T1DM and T2DM mice led to significant improvements in glucose tolerance. In T1DM, semaglutide led to reduced fasting glucose and demonstrated a 21% reduction in peak glucose with a 32.2% decrease in total area under the curve (AUC) compared to untreated T1DM mice (*p* < 0.001; Figure 1C). In T2DM, treated mice showed similar peak glucose levels to the untreated T2DM cohort, but a more rapid return toward baseline values compared to untreated T2DM mice, resulting in a 21.5% reduction in AUC and indicating improved glycemic control (*p* < 0.001; Figure 1D).

### 3.2 Surgical and Infectious Outcomes

#### 3.2.1 Bioluminescence Imaging (BLI) of Infection Burden

**FIGURE 2:**
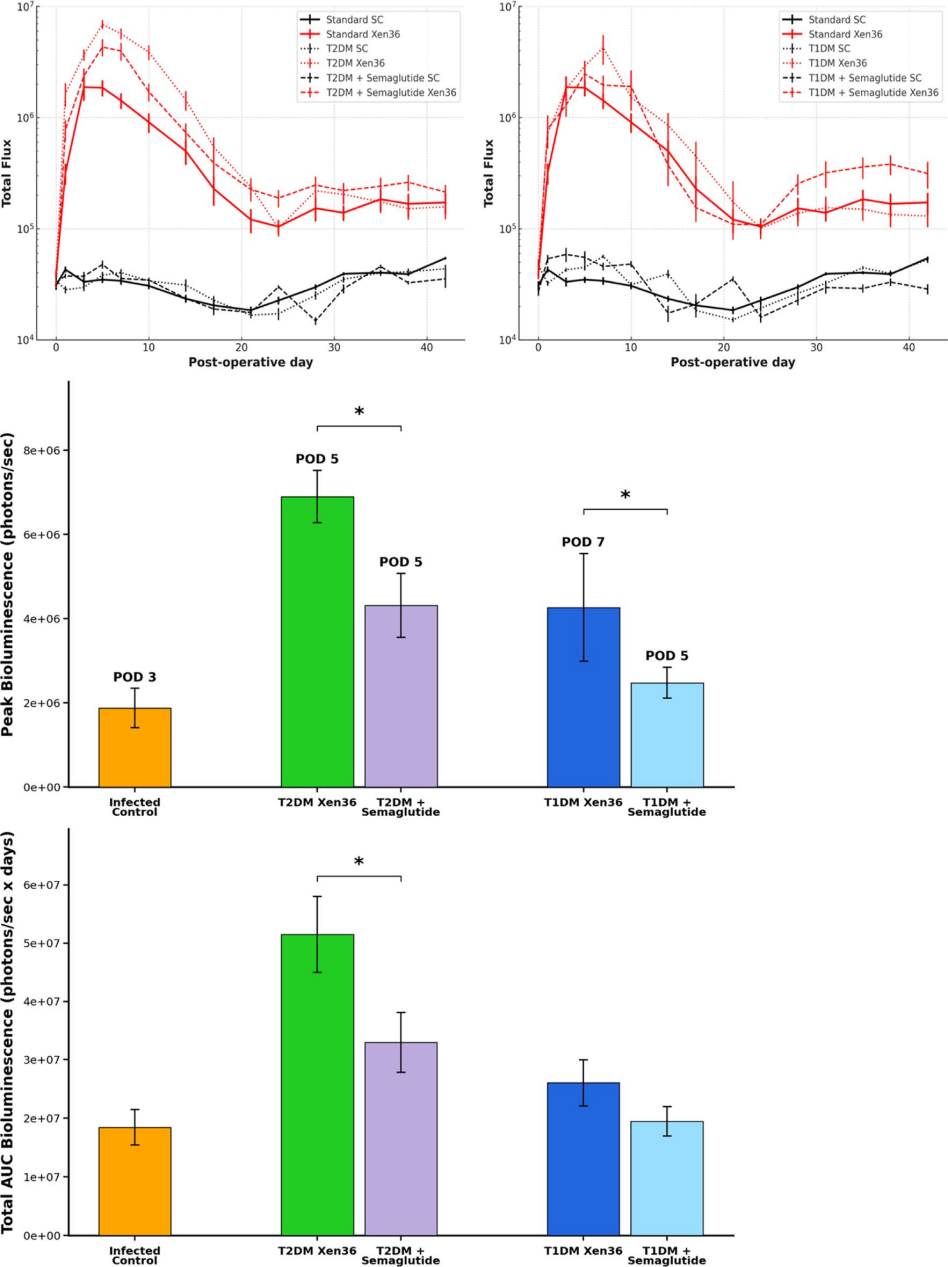
Longitudinal and peak *S. aureus* infectious burden by bioluminescence imaging (BLI). Longitudinal bioluminescence imaging demonstrating infection burden of sterile control (SC) and bioluminescent *S. aureus* (Xen36) inoculated mice following spinal implant surgery in non‐diabetic (standard), Type 2 diabetic (T2DM), Type 1 diabetic (T1DM), and semaglutide‐treated groups. Peak BLI was reached on postoperative day (POD) 3 in controls (1.88 ± 0.47 E6 photons/s), POD 5 in T2DM (6.90 ± 0.62 E6 photons/s) and T2DM + semaglutide (4.31 ± 0.76 E6 photons/s), and POD 7 in T1DM (4.27 ± 1.28 E6 photons/s), with T1DM + semaglutide peaking on POD 5 (2.48 ± 0.37 E6 photons/s). Peak BLI was significantly higher in both diabetic groups compared to controls (*p* < 0.001). Semaglutide reduced peak BLI by 38% in T2DM (*p* < 0.001) and 42% in T1DM (*p* < 0.001) compared to untreated diabetic counterparts. By POD 24, bioluminescence stabilized to a low‐level plateau (~E5 photons/s) across all infected groups. Total BLI AUC was greatest in T2DM, followed by T1DM and control (*p* < 0.001). Semaglutide reduced total BLI AUC by 37% in T2DM (*p* = 0.009) and 24% in T1DM (*p* = 0.233). Data represented as mean ± SD. N: Sterile control = 10, infected control = 10, T2DM sterile = 10, T2DM infected = 27, T2DM + semaglutide sterile = 5, T2DM + semaglutide infected = 15, T1DM sterile = 10, T1DM infected = 27, T1DM + semaglutide sterile = 5, T1DM + semaglutide infected = 15.

Both T2DM and T1DM mice demonstrated marked increases in *S. aureus* infectious burden compared to controls, as measured by bioluminescence (BLI). T2DM mice reached the highest peak BLI on postoperative day 5 (6.90 ± 0.62 E6 photons/s), while T1DM mice exhibited a delayed and slightly lesser peak BLI of 4.27 ± 1.28 E6 photons/s on postoperative day 7 (*p* < 0.001; Figure 2). Both diabetic groups had significantly higher peak infection burdens than controls (1.88 ± 0.47 E6 photons/s on POD 3; *p* < 0.001; Figure 2). T2DM mice also showed a more sustained elevation in BLI, resulting in the greatest AUC, followed by T1DM and then control nondiabetic mice (*p* < 0.001; Figure 2). By postoperative day 24, resolution of the acute inflammatory phase was observed in all infected groups, with bioluminescence stabilizing to a similar, low‐level plateau (E5 photons/s) for the remainder of the study (Figure 2). In all uninfected, control sterile groups, a similar pattern of background BLI was observed at stable levels (E4 photons/s) throughout the duration of the study.

Among semaglutide‐treated groups, a reduction in peak BLI was observed for both T2DM and T1DM mice compared to untreated diabetic cohorts. Among the T2DM semaglutide‐treated group, there was a reduction in peak BLI by approximately 38% (4.31 ± 0.76 E6 photons/s on POD 5; *p* < 0.001; Figure 2) and a 37% reduction in total BLI AUC (*p* = 0.009; Figure 2). Within the T1DM semaglutide‐treated group, there was a 42% reduction in peak BLI (2.48 ± 0.37 E6 photons/s on POD 5; *p* < 0.001; Figure 2) and a 24% decrease in total BLI AUC, though this did not reach statistical significance (*p* = 0.233; Figure 2).

#### 3.2.2 Colony‐Forming Units (CFU)

Median tissue CFU at 6 weeks postoperatively was greatest in the T2DM infected group (145 798 CFU/g), followed by T1DM infected (16 605 CFU/g) and infected controls (6778 CFU/g), while all sterile control groups had a median tissue CFU of zero. For implant‐associated CFU, the highest median was observed in the T1DM infected group (32.3 CFU/implant), with lower medians in T2DM infected (8.3 CFU/implant) and infected control animals (6.3 CFU/implant), and zero in all sterile groups. However, these differences in median CFU among the infected groups were not statistically significant for either tissue (*p* = 0.60) or implant samples (*p* = 0.61), as assessed by Kruskal–Wallis testing.

#### 3.2.3 Wound Dehiscence and Survival Analysis

**FIGURE 3:**
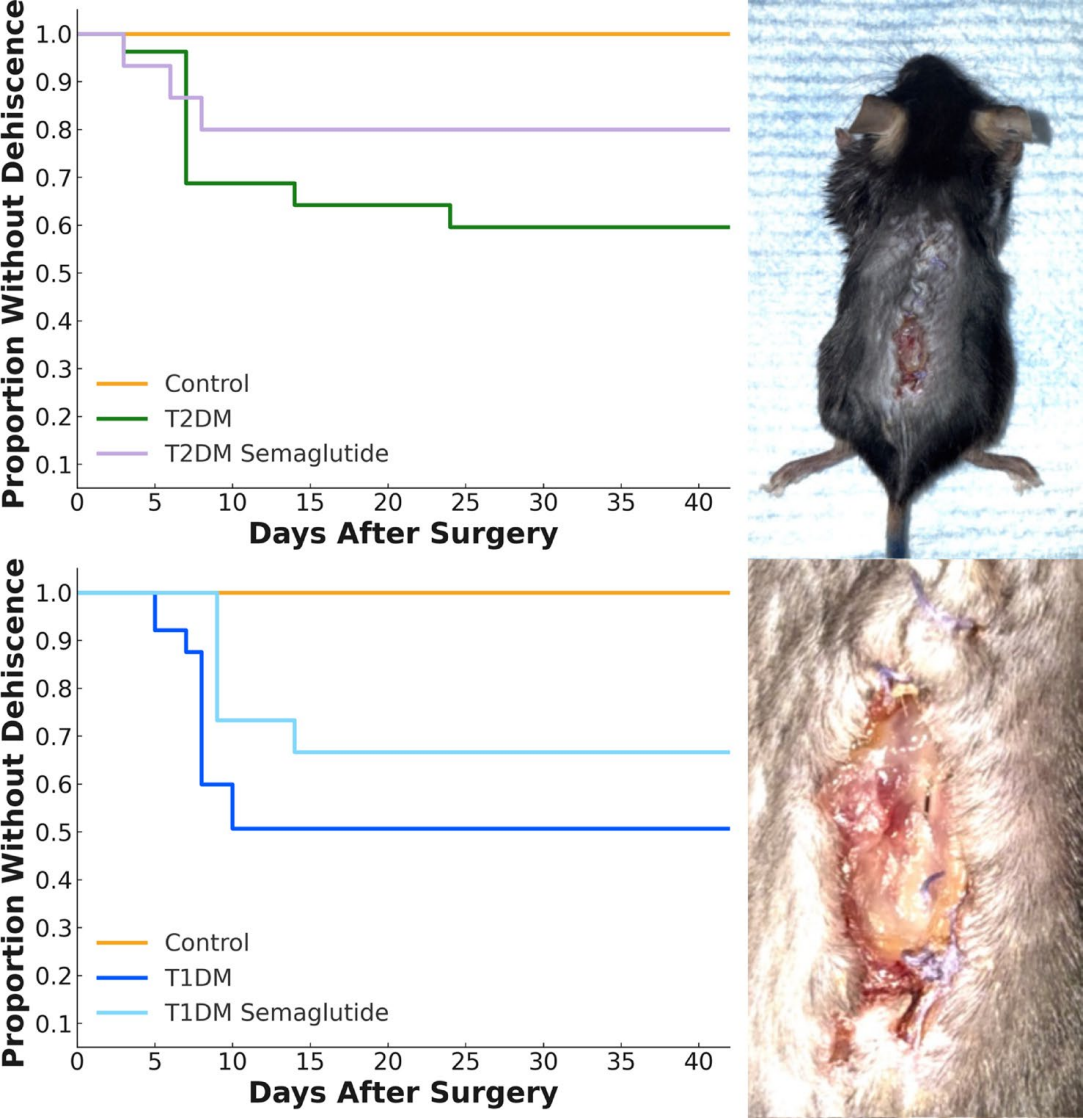
Kaplan–Meier analysis and clinical photographs of wound dehiscence. Representative clinical photograph of a type 1 diabetic (T1DM) mouse demonstrating postoperative wound dehiscence around the spinal implant on postoperative day 7 (POD 7). Kaplan–Meier survival curves illustrate time to wound dehiscence across experimental groups. No wound dehiscence events were observed in nondiabetic controls. Both Type 2 diabetic (T2DM) and T1DM groups experienced significantly higher rates of wound dehiscence compared to controls (*p* < 0.001). T1DM mice demonstrated the earliest loss of wound integrity, with rapid dehiscence between POD 5 and 10, while T2DM mice exhibited intermediate rates between POD 7 and 24. Semaglutide reduced overall wound dehiscence by 40% in T2DM (*p* = 0.273) and 19% in T1DM (*p* = 0.213) compared to untreated diabetic counterparts, though these differences did not reach statistical significance on log‐rank survival analysis. N: Infected control = 10, T2DM infected = 27, T2DM + semaglutide infected = 15, T1DM infected = 27, T1DM + semaglutide infected = 15.

Wound healing complications were common in diabetic groups. Kaplan–Meier analysis of wound dehiscence revealed no events in the nondiabetic control group, who maintained intact wounds throughout the observation period, while both T2DM and T1DM groups experienced early and frequent dehiscence compared to control (*p* < 0.001; Figure 3). T1DM mice had the worst wound survival, with rapid loss of wound integrity between postoperative days 5 and 10. T2DM mice showed intermediate rates and timing of dehiscence between postoperative days 7 and 24 (Figure 3). Semaglutide decreased the overall occurrence of wound dehiscence in T1DM and T2DM cohorts by 19% and 40%, respectively, although these findings do not reach significance on survival analysis (*p* = 0.213 and *p* = 0.273; Figure 3).

### 3.3 Immune Response and Inflammatory Differences

#### 3.3.1 Immunologic and Cytokine Profiles

**FIGURE 4:**
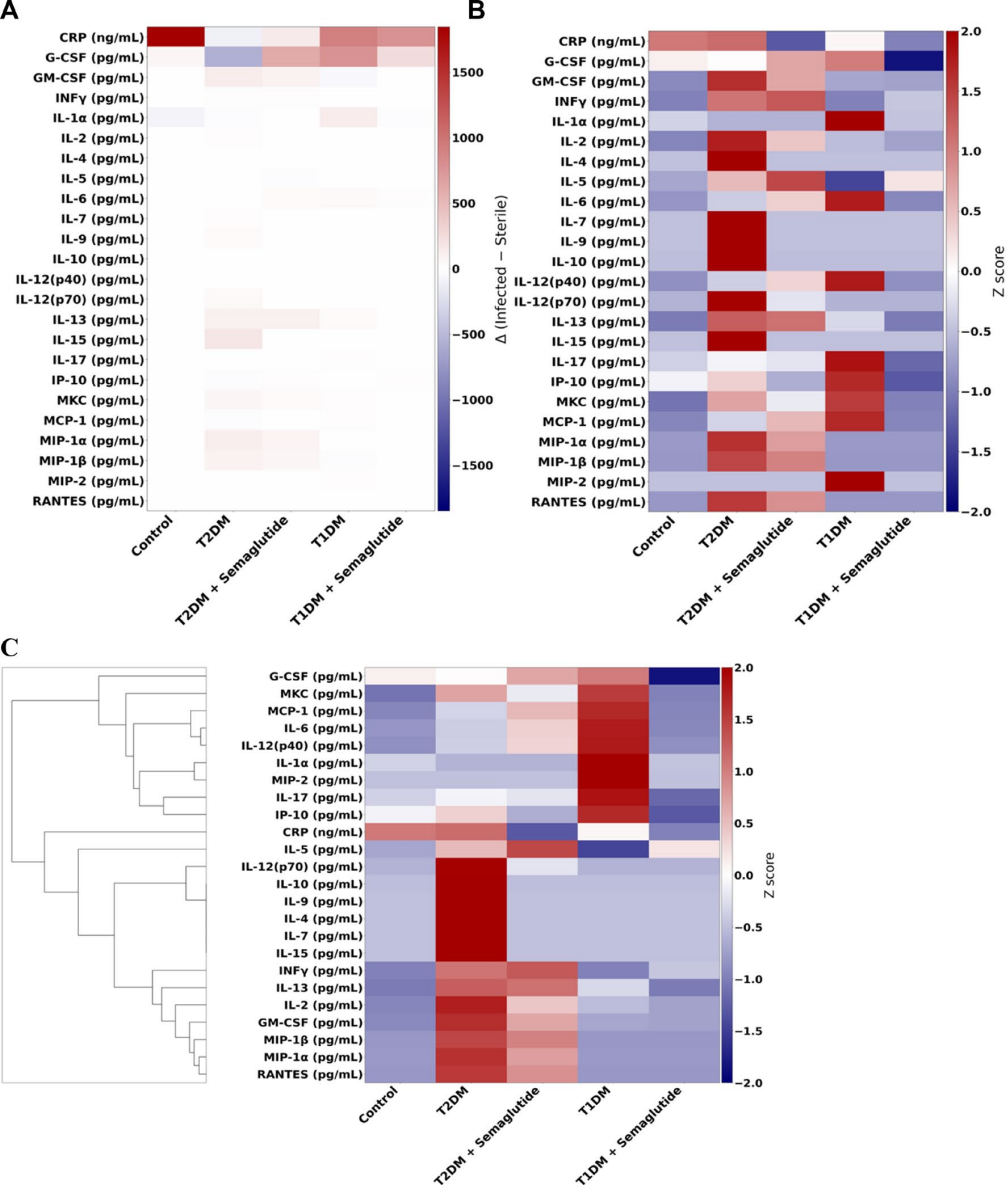
Circulating cytokine profile heat maps and cluster analyzes. (A) Absolute heat map displaying differential serum cytokine concentrations between sterile and infected animals across experimental groups: Nondiabetic (control), Type 2 diabetic (T2DM), Type 1 diabetic (T1DM), and semaglutide‐treated groups. (B) Normalized Z‐score relative heat map illustrating changes in cytokine levels between sterile and infected conditions for each group. (C) Cluster analysis of differential cytokine expression patterns, grouped by similarity across experimental conditions. In nondiabetic controls, mean CRP increased from 6833 ng/mL (sterile) to 8680 ng/mL (infected), with proportional increases in IL‐6 (+64%), G‐CSF (+52%), and IP‐10 (+49%). T2DM mice demonstrated elevated baseline cytokine levels with minimal change following infection (< 10% variation across most analytes). T1DM mice exhibited lower baseline levels with large proportional increases after infection (IL‐6 + 73%, IL‐17 + 58%, IFN‐γ +64%, G‐CSF +47%). Semaglutide reduced IL‐6, MCP‐1, and TNF‐α by 24%–32% in T2DM and IL‐6 and IFN‐γ by 35% and 41% in T1DM, respectively, while approximately doubling IL‐10 levels in both diabetic cohorts. N: Sterile control = 3, infected control = 3, T1DM sterile = 4, T1DM infected = 5, T1DM + semaglutide sterile = 4, T1DM + semaglutide infected = 5, T2DM sterile = 3, T2DM infected = 5, T2DM + semaglutide sterile = 4, T2DM + semaglutide infected = 5.

Quantitative cytokine analysis demonstrated group‐specific differences in systemic inflammatory activity under both sterile and infected conditions (Figure 4). In nondiabetic controls, mean CRP increased from 6833 ng/mL in sterile mice to 8680 ng/mL after infection (Δ = +1847 ng/mL). Similar proportional increases were observed in IL‐6 (+64%), G‐CSF (+52%), and IP‐10 (+49%) following bacterial inoculation.

T2DM mice displayed the highest baseline circulating cytokine concentrations and minimal postinfection changes. Baseline CRP measured 8917 ng/mL and decreased slightly to 8814 ng/mL after infection (Δ = −103 ng/mL). IL‐6, TNF‐α, and MCP‐1 remained elevated at baseline (2.7–3.2× control levels) but changed less than 10% following inoculation. G‐CSF, GM‐CSF, and IP‐10 showed similarly limited variation, indicating a stable cytokine profile across conditions. T1DM mice exhibited lower baseline cytokine concentrations but greater proportional changes after infection. Mean CRP increased from 6535 to 7450 ng/mL (Δ = +915 ng/mL). IL‐6 increased by 73%, IL‐17 by 58%, IFN‐γ by 64%, and G‐CSF by 47% relative to sterile T1DM levels. MCP‐1 and GM‐CSF also rose modestly (+28% and +19%, respectively).

Semaglutide administration reduced baseline cytokine levels in both diabetic models. In T2DM mice, IL‐6 decreased by 32%, MCP‐1 by 29%, and TNF‐α by 24% compared with untreated T2DM values, while infection produced modest increases in G‐CSF (+22%) and IL‐10 (+31%). In T1DM mice, IL‐6 and IFN‐γ were 35% and 41% less, respectively, than untreated T1DM, and IL‐10 increased approximately twofold. Across all measured analytes, semaglutide‐treated groups showed intermediate absolute cytokine concentrations and distributions between untreated diabetic and nondiabetic control values.

#### 3.3.2 Tissue Immunofluorescence and Histology

**FIGURE 5:**
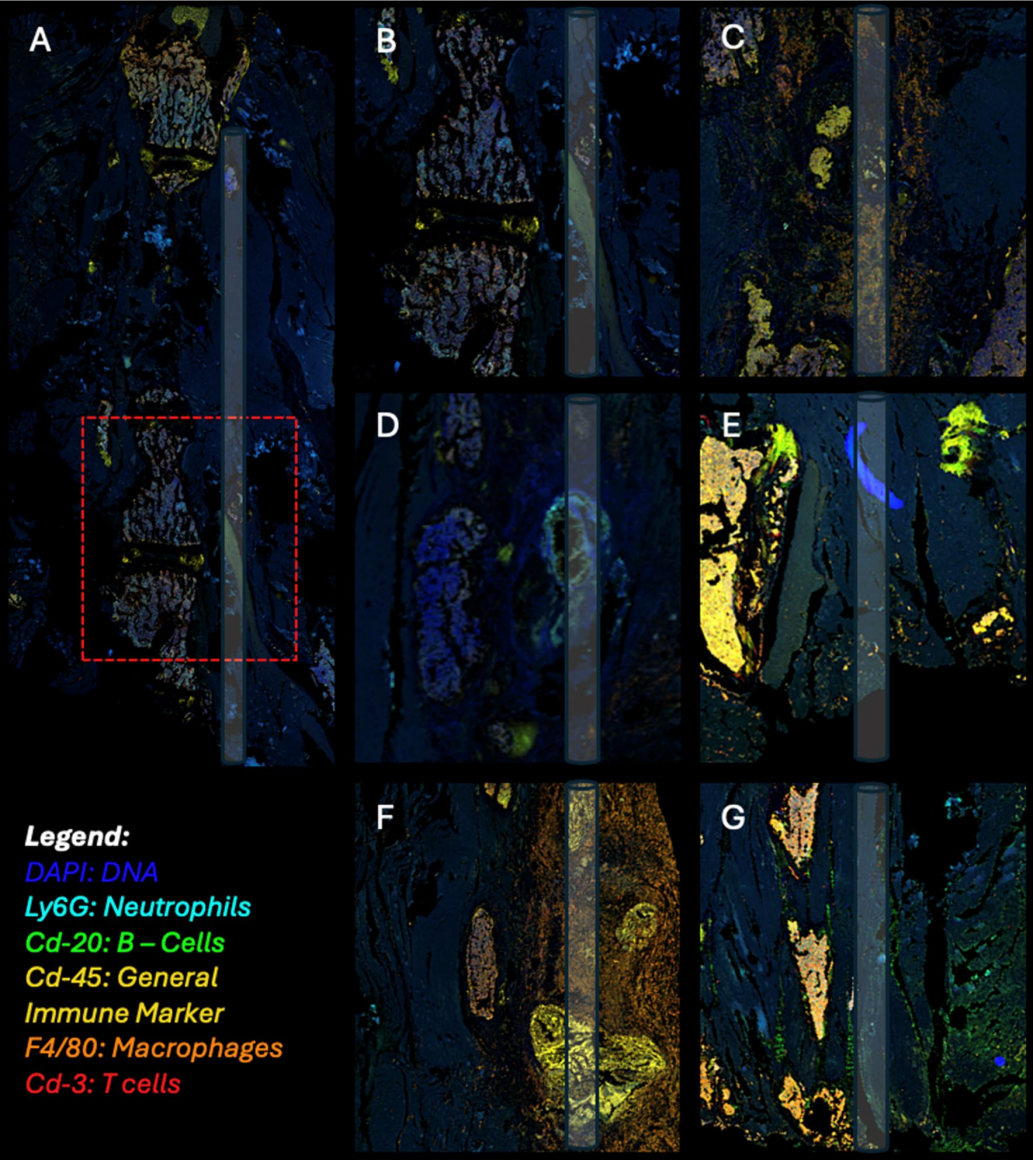
Immunofluorescent staining of paraspinal tissues on postoperative day 42 (POD 42). Representative immunofluorescent images demonstrate spatial localization and intensity of immune cell infiltrates across experimental groups at study endpoint (POD 42). (A) shows a low‐magnification sterile control spine highlighting the region of interest (red dashed box). (B) Displays a higher‐magnification view of the sterile control, showing minimal immune cell presence. Panels (C–G) show infected control, Type 2 diabetic (T2DM), T2DM + semaglutide, Type 1 diabetic (T1DM), and T1DM + semaglutide groups, respectively, revealing variable degrees of inflammatory infiltration. T2DM infected tissues (D) demonstrated broad Ly6G+ neutrophil and F4/80+ macrophage co‐localization with attenuated CD3+ T‐cell and CD20+ B‐cell signals. Semaglutide‐treated T2DM tissues (E) exhibited marked reduction in immune fluorescence intensity with sparse residual staining limited to peri‐implant regions and increased collagenous organization. T1DM infected tissues (F) showed dense, heterogeneous infiltrates comprising CD3+ T cells, CD20+ B cells, and Ly6G+ neutrophils diffusely distributed throughout paraspinal soft tissue. Semaglutide‐treated T1DM tissue (G) demonstrated reduced immune density with predominance of CD45+ and F4/80+ cells in a fibrotic matrix, consistent with resolution‐phase remodeling. Multichannel staining identifies nuclei (DAPI, blue), neutrophils (Ly6G, cyan), B‐cells (CD20, green), general immune cells (CD45, yellow), macrophages (F4/80, orange), and T‐cells (CD3, red). Images are representative of *n* = 2–3 animals analyzed per cohort.

Multiplex immunofluorescence of paraspinal tissues demonstrated distinct immune cell distribution patterns across diabetic and non‐diabetic groups (Figure 5). Sterile control tissues (Figure 5A,B) showed organized muscle and fibrous architecture with minimal CD45^+^ immune cell presence, confirming baseline quiescence. Infected control tissues (Figure 5C) exhibited increased cellular infiltration localized around the implant tract, with moderate accumulation of CD45^+^ leukocytes and F4/80^+^ macrophages, accompanied by scattered Ly6G^+^ neutrophils. T2DM infected tissues (Figure 5D) showed broad areas of Ly6G^+^ neutrophil and F4/80^+^ macrophage co‐localization, indicative of persistent neutrophilic inflammation. These findings were accompanied by attenuated CD3^+^ T‐cell and CD20^+^ B‐cell signals, reflecting impaired recruitment of the adaptive immune response. Notably, semaglutide‐treated T2DM tissues (Figure 5E) exhibited marked reduction in overall immune fluorescence intensity, with sparse residual immune cell staining limited to peri‐implant regions and increased collagenous organization. In contrast, T1DM mice (Figure 5F), infection produced dense and heterogeneous immune infiltrates comprising CD3^+^ T cells, CD20^+^ B cells, and Ly6G^+^ neutrophils diffusely distributed throughout the paraspinal soft tissue. The spatial pattern was consistent with an acute‐on‐chronic inflammatory response. Semaglutide‐treated T1DM tissue (Figure 5G) demonstrated reduced immune density, with a predominance of CD45^+^ and F4/80^+^ cells in a fibrotic matrix, suggesting resolution‐phase remodeling rather than ongoing acute inflammation.

**FIGURE 6:**
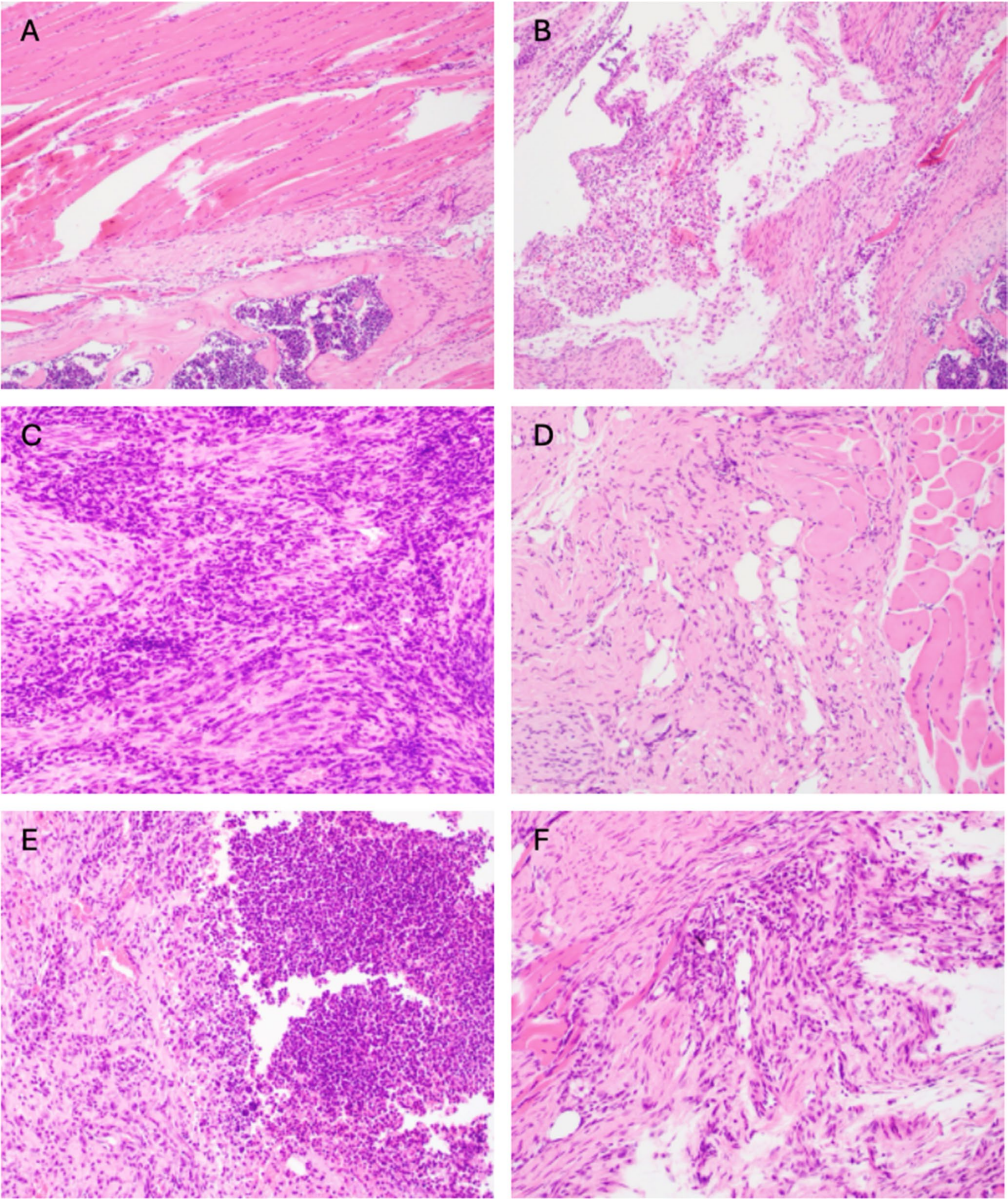
Representative histologic images of paraspinal tissues from sterile and *S. aureus* infected groups in diabetic and nondiabetic mice on postoperative day 42 (POD 42). Hematoxylin and eosin–stained sections (10× or 20× magnification) demonstrate variable inflammatory responses across experimental groups. Sterile control tissue (A) shows surgical wound‐induced focal scar formation without significant inflammation. Infected control tissue (B) demonstrates scar formation with moderate lympho‐histiocytic inflammation and rare neutrophils. Type 2 diabetic (T2DM) infected tissue (C) reveals extensive mixed inflammation predominantly of neutrophils with lymphocytes and histiocytes in a fibrotic background, while semaglutide‐treated T2DM tissue (D) demonstrates predominantly fibrotic tissue with rare inflammatory cells, indicating attenuation of infection. Type 1 diabetic (T1DM) infected tissue (E) exhibits marked mixed inflammation with neutrophils, lymphocytes, and histiocytes, whereas semaglutide‐treated T1DM tissue (F) shows fibrosis and scar formation with mild to moderate inflammation composed of lymphocytes, histiocytes, and rare neutrophils. Images are representative of *n* = 2–3 animals analyzed per cohort.

Histologic evaluation by hematoxylin and eosin staining (Figure 6) corroborated the immunofluorescence findings. Sterile control tissues from uninfected animals (Figure 6A) demonstrated organized muscle and fibrotic postsurgical repair without significant inflammation. Infected controls (Figure 6B) showed mild to moderate lympho‐histiocytic inflammation with focal neutrophil aggregates. T2DM infected tissues (Figure 6C) were characterized by extensive disorganized fibrosis and dense sheets of neutrophils intermixed with lymphocytes and histiocytes, consistent with sustained inflammation, while semaglutide‐treated T2DM tissues (Figure 6D) exhibited predominantly fibrotic stroma with minimal inflammatory cell presence, indicating attenuation of infection and progression toward tissue repair. T1DM infected tissues (Figure 6E) revealed robust mixed inflammatory infiltrates including neutrophils, lymphocytes, and histiocytes, while semaglutide‐treated T1DM sections (Figure 6F) displayed maturing fibrotic tissue with reduced cellularity and only mild residual inflammation.

## 4 Discussion

This study demonstrates that both T2DM and T1DM markedly increase susceptibility to spinal implant–associated *S. aureus* infection and impair wound healing in a murine model despite phenotypic differences. Both diabetic models showed greater infectious burden, higher rates of wound dehiscence, and profound alterations in systemic cytokine and tissue inflammatory profiles compared with nondiabetic controls. While these findings in this study suggest differing inflammatory phenotypes of T2DM and T1DM, they also identify metabolic modulation with semaglutide as a potential beneficial modifier of both these disease‐specific immune responses.

### 4.1 Infection Severity and Wound Complications

Consistent with prior studies of orthopedic and spine infection, diabetes markedly worsened infection severity and delayed wound healing [26, 27]. Both T2DM and T1DM cohorts exhibited higher bacterial bioluminescence signals, elevated cumulative infectious burden, and substantially increased wound dehiscence compared with nondiabetic controls (Figure 2). T2DM mice showed the highest and earliest infection peaks and sustained elevations in bacterial signal over time to ~1‐log higher than infectious control subjects, whereas T1DM mice exhibited a delayed but comparably severe trajectory. Wound survival analysis demonstrated complete integrity in control mice and frequent wound failure in both diabetic groups, with both groups showing high rates of dehiscence compared to controls (Figure 3). These findings reinforce clinical data identifying diabetes as a strong independent risk factor for postoperative infection and wound failure following orthopedic procedures [28, 29].

Semaglutide treatment mitigated these effects across both diabetic models (Figure 2). Treated mice exhibited lower peak infection signals, decreased wound dehiscence, and reduced overall infectious burden relative to untreated diabetic counterparts (Figure 3). These findings indicate that metabolic modulation can lessen the severity and chronicity of postoperative infection in the diabetic state, aligning with observed reductions in systemic and local inflammatory activity.

### 4.2 Distinct Systemic Inflammatory Profiles in T2DM and T1DM

Quantitative circulating cytokine profiling revealed distinct patterns of systemic inflammation between T2DM and T1DM (Figure 4). T2DM mice demonstrated elevated baseline CRP and high levels of IL‐6, TNF‐α, and MCP‐1 even in the absence of *S. aureus* infection, with minimal changes following bacterial challenge (< 10% variation across most cytokines). This pattern indicates chronic activation of inflammatory pathways with limited inducibility after new infectious insult. By contrast, T1DM mice exhibited lower baseline cytokine levels but larger proportional increases following infection, with IL‐6 rising by 73%, IL‐17 by 58%, IFN‐γ by 64%, and G‐CSF by 47%. These data define two distinct systemic inflammatory states: a persistently elevated but unresponsive milieu in T2DM, and an exaggerated inducible response in T1DM. These findings corroborate prior research which demonstrates a diminished immunoglobulin response in a similar T2DM murine model of periprosthetic joint infection [30]. Furthermore, these findings parallel clinical observations of elevated baseline inflammation in patients with metabolic syndrome and T2DM, and suggest that chronic inflammation in T2DM may paradoxically impair the host's ability to generate effective responses to new infectious insults that may reflect immune “exhaustion” or a ceiling effect [31, 32].

Semaglutide administration altered both baseline and post‐infection cytokine profiles (Figure 4). In T2DM mice, semaglutide reduced IL‐6, MCP‐1, and TNF‐α by 24%–32%, while increasing infection‐associated G‐CSF and IL‐10. In T1DM, semaglutide lowered IL‐6 and IFN‐γ by 35% and 41%, respectively, and approximately doubled IL‐10 levels. Cluster analysis positioned semaglutide‐treated cohorts between untreated diabetic and nondiabetic controls, indicating partial normalization of systemic cytokine patterns. Together, these results suggest that semaglutide mitigates both chronic inflammatory priming in the immune‐exhausted T2DM mice and excessive cytokine activation in T1DM mice, producing a more regulated systemic inflammatory state in both cohorts.

### 4.3 Local Immune and Tissue‐Level Responses

Local analyzes reflected the systemic immune differences between diabetic subtypes (Figures 5 and 6). In nondiabetic controls, *S. aureus* infection produced organized fibrotic repair with limited immune infiltration, consistent with a contained and resolving post‐surgical response. Both diabetic models showed broader, disorganized inflammation, but with distinct cellular patterns.

T2DM tissues were characterized by neutrophil and macrophage infiltration, indicative of persistent neutrophilic inflammation, as well as an attenuated adaptive immune response. Similar inflammatory findings were observed in the femur of a T2DM murine model of periprosthetic joint infection [33]. T1DM tissues demonstrated diffuse, mixed immune infiltrates with abundant CD3^+^, CD20^+^, Ly6G^+^, and F4/80^+^ cells, accompanied by histologic evidence of persistent inflammation and incomplete fibrosis. This pattern suggests an exaggerated but ineffective response that parallels prolonged wound inflammation and delayed infectious resolution observed in this study [34, 35].

Semaglutide treatment reduced inflammatory cell density and improved tissue organization in both diabetic models. Tissues from semaglutide‐treated mice showed less neutrophilic persistence and more mature fibrosis, suggesting improved inflammatory resolution. Clinically, these findings imply that metabolic modulation through the GLP‐1 receptor can help restore coordinated local immune activity and promote more effective wound repair in diabetic surgical sites.

### 4.4 Translational Implications and Clinical Relevance

Taken together, the findings from this study suggest that T2DM and T1DM demonstrate distinct phenotypes in the setting of spinal implant‐associated infection. These phenotypes are characterized by chronic inflammatory saturation with a blunted acute response and exaggerated immune activation with inadequate immune regulation, respectively. Although the underlying phenotypes differ, both culminate in impaired clearance of bacterial challenge and increased wound complications perioperatively. This study is the first to detail such relationships with respect to spine surgery.

In the present study, restoration of metabolic and inflammatory balance with semaglutide improved glucose tolerance and decreased the staphylococcal infection burden, while modulating cytokine and tissue inflammatory responses in both T2DM and T1DM models. These results align with prior preclinical reports showing that GLP‐1 signaling enhances neutrophil function, reduces oxidative stress, and limits macrophage activation in a diabetic murine model [36]. While early human data and database studies on this subject are promising, this is a hypothesis that warrants further investigation in both preclinical and clinical settings [37, 38, 39]. This study is the first longitudinal in vivo study detailing such effects of semaglutides in the perioperative setting of spine surgery. This study also supports literature suggesting that semaglutide may have a beneficial effect in T1DM subjects [19, 20].

Although semaglutide shifted systemic cytokine profiles and tissue inflammation toward a nondiabetic‐like state by partially normalizing infection burden and wound healing outcomes in both diabetic subtypes, the effect was not complete in either, indicating that altering metabolic control alone does not fully restore host defense. The observation that GLP‐1 receptor activation improves infection outcomes in both T2DM and T1DM, despite fundamentally distinct primary disease patterns, suggests that there exists a common downstream GLP‐1–responsive pathway representing a potential convergent therapeutic target to mitigate implant‐associated infection in T2DM and T1DM patients perioperatively.

### 4.5 Strengths, Limitations, and Future Directions

Strengths of this study include the use of well‐validated murine models, longitudinal in vivo tracking of infection, and comprehensive immunophenotyping. Limitations include the potential for strain‐specific differences in immune response, the use of a single pathogen, and the challenge of fully recapitulating human diabetic physiology in mice. This will partly be addressed in subsequent studies evaluating bacterial strain‐specific outcomes. Importantly, while our T2DM model reliably produces hyperglycemia and obesity typical of metabolic syndrome, we were unable to control for obesity as an independent variable. As such, the effects observed in T2DM animals may be exacerbated by obesity and related metabolic disturbances, making it difficult to fully disentangle the contributions of hyperglycemia, insulin resistance, and obesity to infection risk and immune dysfunction. Nonetheless, these same confounders influence guidelines in human subjects, suggesting our efforts to phenocopy human pathology in mice were relatively successful. The STZ model for T1DM was effective and practical for use in this study, though it is one of many T1DM murine models. Future studies should explore validation of these results with T1DM models such as NOD mice. Furthermore, this study provides qualitative tissue‐level responses, and future directions should include quantification of immune response within the tissues, and the role of implant‐bone integration and osteolysis. Lastly, tissue and hematologic data were only collected at a single time point, reflecting only representative snapshots of end‐stage inflammatory states. Future work will focus on dissecting cellular immune function in more detail and at more time points, exploring the role of semaglutide in T1DM mice, and extending these findings to models of spinal fusion and pseudarthrosis.

## 5 Conclusion

In summary, both T2DM and T1DM substantially impair host defense against *S. aureus* spinal implant infection, albeit with distinct systemic and local inflammatory patterns. T2DM is associated with elevated baseline inflammation and reduced acute responsiveness, whereas T1DM produces exaggerated cytokine induction with inefficient infection resolution. T2DM is often associated with obesity/metabolic syndrome, which may exacerbate these findings. Semaglutide improved glycemic control, lowered inflammatory burden, reduced infectious complications such as wound dehiscence, and normalized the immune response both serologically and histologically across both diabetic models. These results support further evaluation of metabolic modulation through GLP‐1R activation as a strategy to optimize surgical outcomes in all diabetic patients.

## Author Contributions

**Joshua Mehany:** investigation. **Autreen Golzar:** investigation, validation, methodology, formal analysis, data curation. **Trevor S. Lloyd:** investigation, methodology, formal analysis, project administration, data curation, validation, writing – original draft. **Soroush Shahamatdar:** investigation. **Lauren Pearce:** investigation. **Joshua Wiener:** investigation, methodology, validation, formal analysis, data curation. **Andrew Kittredge:** investigation. **Kevin P. Francis:** investigation, methodology, validation, project administration, supervision. **Christopher D. Hamad:** investigation, methodology. **Thomas E. Olson:** conceptualization, investigation, funding acquisition, writing – original draft, methodology, validation, visualization, writing – review and editing, formal analysis, data curation. **Rene F. Chun:** investigation, methodology, validation, formal analysis, project administration, data curation, supervision. **William L. Sheppard:** conceptualization, investigation, funding acquisition, methodology, validation, visualization, writing – review and editing, project administration, supervision, resources. **Langston T. Holly:** investigation, validation, project administration, supervision. **Nicholas M. Bernthal:** supervision, resources, validation, methodology, writing – review and editing, conceptualization, investigation, funding acquisition. **Michael R. Yeaman:** investigation, validation, methodology, supervision, project administration. **John S. Adams:** conceptualization, investigation, funding acquisition, writing – review and editing, methodology, validation, supervision, resources. **Farres Obeidin:** investigation, methodology, validation, project administration, supervision, data curation.

## Funding

This work was supported by the National Institutes of Health (AR059033).

## Disclosure

Large language models were utilized solely to assist with language refinement, sentence structure, and overall organization of the manuscript. All scientific content, including data, analyzes, interpretations, and conclusions, was independently developed, verified, and approved by the authors.

## Conflicts of Interest

The authors declare no conflicts of interest.

## Supporting information


**Figure S1:** Microcomputed tomography (microCT) reconstruction and anterior–posterior and lateral radiographs of the murine spinal implant model. Representative noninvasive postoperative images demonstrating placement of the custom L‐shaped stainless‐steel implant spanning the posterior elements of the lumbar spine. The implant is seated through the spinous processes at the L3–L4 levels with distal extension along the paraspinal surface. This is a representative example of consistent implant positioning across experimental groups and serves as an anatomic reference for subsequent bioluminescence imaging, radiography, histology, and immunofluorescence analyzes.
**Figure S2:** Longitudinal bioluminescence imaging of *S. aureus* spinal implant–associated infection across experimental groups. Representative in vivo bioluminescence images are shown for nondiabetic controls (CT), Type 1 diabetes (T1D), Type 1 diabetes treated with semaglutide (T1D + S), Type 2 diabetes (T2D), and Type 2 diabetes treated with semaglutide (T2D + S) on postoperative days (POD) 1, 3, 7, 21, and 42. Each panel depicts two mice from the respective group at each time point, with luminescent signal intensity corresponding to bacterial burden at the surgical site.

## Data Availability

The data that support the findings of this study are available from the corresponding author upon reasonable request.
