# Supplementary material for: Spinal Implant–Associated Infection in Type 2 and Type 1 Diabetes: Phenotype‐Specific Inflammatory Features and Therapeutic Response to Semaglutide
Source: JOR Spine. 2026 May 28;9(2):e70196. doi: 10.1002/jsp2.70196 (PMC13239351; doi:10.1002/jsp2.70196)
Supplement: Supplementary file 1 — Figure S1: Microcomputed tomography (microCT) reconstruction and anterior–posterior and lateral radiographs of the murine spinal implant model. Representative noninvasive postoperative images demonstrating placement of the custom L‐shaped stainless‐steel implant spanning the posterior elements of the lumbar spine. The implant is seated through the spinous processes at the L3–L4 levels with distal extension along the paraspinal surface. This is a representative example of consistent implant positioning across experimental groups and serves as an anatomic reference for subsequent bioluminescence imaging, radiography, histology, and immunofluorescence analyzes. Figure S2: Longitudinal bioluminescence imaging of S. aureus spinal implant–associated infection across experimental groups. Representative in vivo bioluminescence images are shown for nondiabetic controls (CT), Type 1 diabetes (T1D), Type 1 diabetes treated with semaglutide (T1D + S), Type 2 diabetes (T2D), and Type 2 diabetes treated with semaglutide (T2D + S) on postoperative days (POD) 1, 3, 7, 21, and 42. Each panel depicts two mice from the respective group at each time point, with luminescent signal intensity corresponding to bacterial burden at the surgical site. [file JSP2-9-e70196-s001.docx]

**Supplemental Materials**


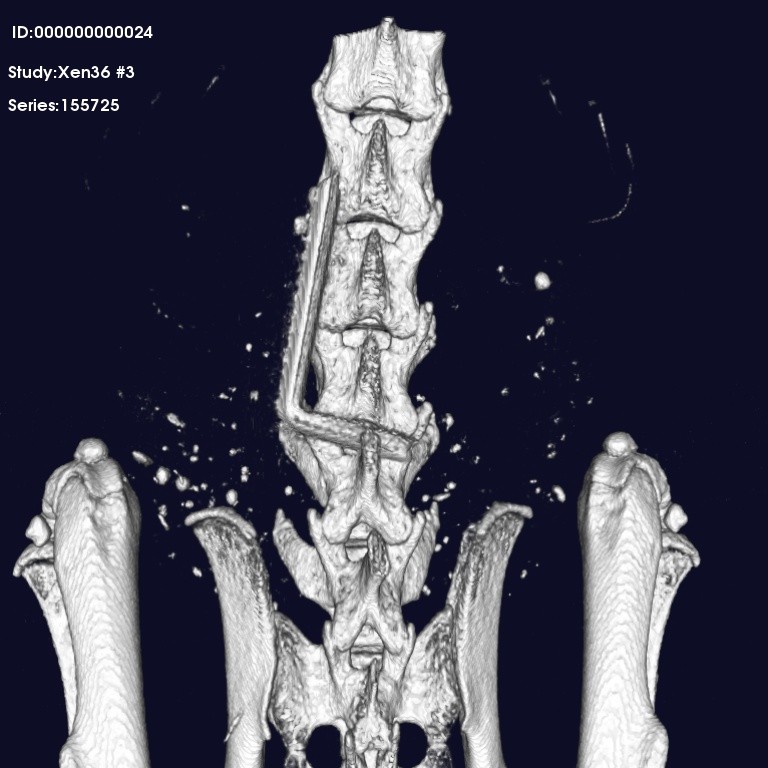

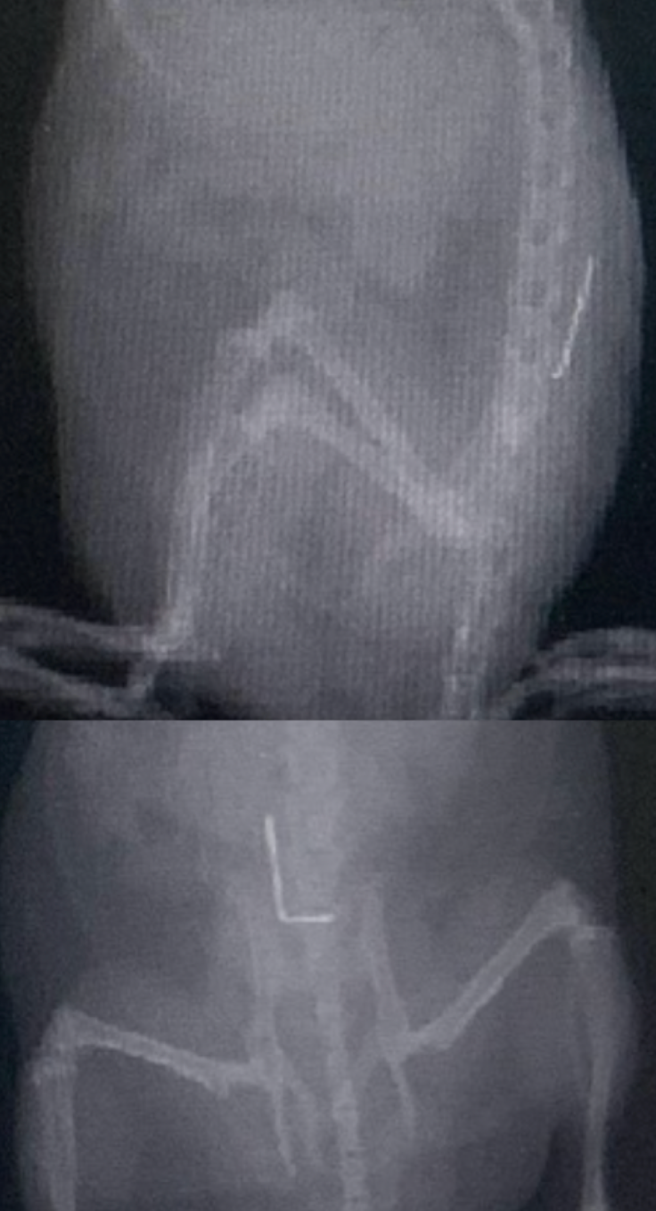


**Supplemental Figure 1. Micro–computed tomography (microCT) reconstruction and anterior-posterior and lateral radiographs of the murine spinal implant model.** Representative non-invasive postoperative images demonstrating placement of the custom L-shaped stainless-steel implant spanning the posterior elements of the lumbar spine. The implant is seated through the spinous processes at the L3–L4 levels with distal extension along the paraspinal surface. This is a representative example of consistent implant positioning across experimental groups and serves as an anatomic reference for subsequent bioluminescence imaging, radiography, histology, and immunofluorescence analyses.


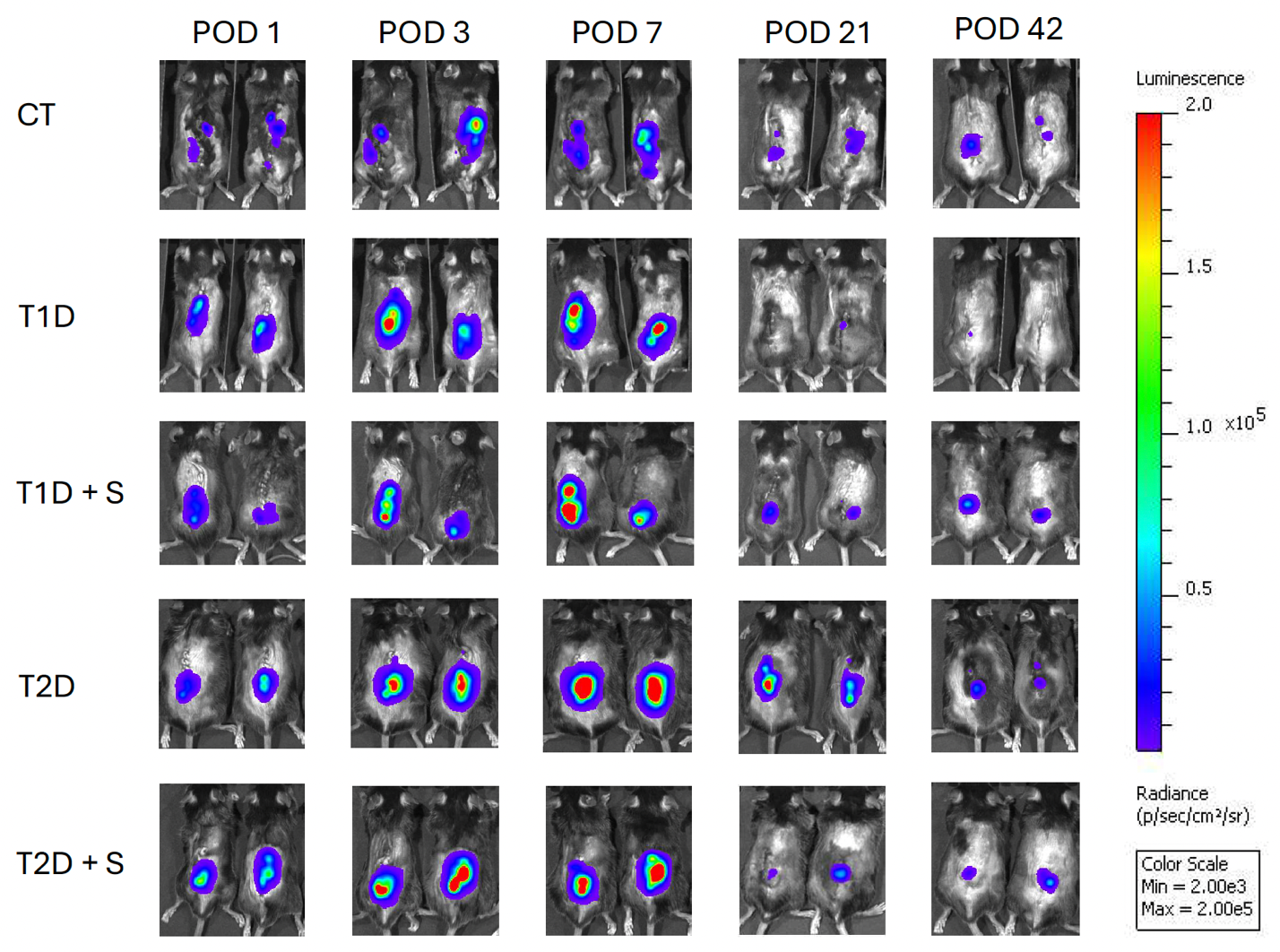


**Supplemental Figure 2. Longitudinal bioluminescence imaging of *S. aureus* spinal implant–associated infection across experimental groups.** Representative *in vivo* bioluminescence images are shown for non-diabetic controls (CT), type 1 diabetes (T1D), type 1 diabetes treated with semaglutide (T1D+S), type 2 diabetes (T2D), and type 2 diabetes treated with semaglutide (T2D+S) on postoperative days (POD) 1, 3, 7, 21, and 42. Each panel depicts two mice from the respective group at each time point, with luminescent signal intensity corresponding to bacterial burden at the surgical site.
